# Understanding the Public’s Reservations about Broad Consent and Study-By-Study Consent for Donations to a Biobank: Results of a National Survey

**DOI:** 10.1371/journal.pone.0159113

**Published:** 2016-07-14

**Authors:** Raymond Gene De Vries, Tom Tomlinson, Hyungjin Myra Kim, Chris Krenz, Diana Haggerty, Kerry A. Ryan, Scott Y. H. Kim

**Affiliations:** 1 Center for Bioethics and Social Sciences in Medicine, University of Michigan, Ann Arbor, Michigan, United States of America; 2 Center for Ethics and Humanities in the Life Sciences, Michigan State University, East Lansing, Michigan, United States of America; 3 Center for Statistical Consultation and Research, University of Michigan, Ann Arbor, Michigan, United States of America; 4 Department of Epidemiology and Biostatistics, Michigan State University, East Lansing, Michigan, United States of America; 5 Department of Bioethics, Clinical Center, National Institutes of Health, Bethesda, Maryland, United States of America; 6 Department of Psychiatry, University of Michigan, Ann Arbor, Michigan, United States of America; University of Torino, ITALY

## Abstract

Researchers and policymakers do not agree about the most appropriate way to get consent for the use of donations to a biobank. The most commonly used method is blanket—or broad—consent where donors allow their donation to be used for any future research approved by the biobank. This approach does not account for the fact that some donors may have moral concerns about the uses of their biospecimens. This problem can be avoided using “real-time”—or study-by-study—consent, but this policy places a significant burden on biobanks. In order to better understand the public’s preferences regarding biobank consent policy, we surveyed a sample that was representative of the population of the United States. Respondents were presented with 5 biobank consent policies and were asked to indicate which policies were acceptable/unacceptable and to identify the best/worst policies. They were also given 7 research scenarios that could create moral concern (e.g. research intending to make abortions safer and more effective) and asked how likely they would be to provide broad consent knowing that their donation might be used in that research. Substantial minorities found both broad and study-by-study consent to be unacceptable and identified those two options as the worst policies. Furthermore, while the type of moral concern (e.g., regarding abortion, the commercial use of donations, or stem cell research) had no effect on policy preferences, an increase in the number of research scenarios generating moral concerns was related to an increased likelihood of finding broad consent to be the worst policy. The rejection of these ethically problematic and costly extremes is good news for biobanks. The challenge now is to design a policy that combines consent with access to information in a way that assures potential donors that their interests and moral concerns are being respected.

## Introduction

Biobanks are broadly defined as entities that store biological specimens for clinical purposes or for research. [1] The long-term storage of biological specimens in biobanks has contributed substantially to medical science, providing biomedical researchers access to numbers and varieties of specimens that would otherwise require substantial time and money to assemble. Research using these specimens has improved our understanding of the causes of many diseases, including multiple sclerosis[2], prostate cancer[3], cervical cancer[4, 5], and lung cancer.[6] Biobanking is essential to the work of precision medicine[7], leading to advances in diagnosis and treatment. In 2015, the White House announced the Precision Medicine Initiative (PMI), with the goal of assembling a longitudinal cohort of one million Americans willing to donate biological specimens to be stored in a biobank together with de-identified demographic and health data. [8, 9] If successful, the PMI will greatly expand our knowledge in the fields of genomics, metabolomics, and proteomics.

Most biobank research can be done using this de-identified data, which is stripped of personal identifiers after the initial specimen donation. At the same time, the full range of possible future research uses will be unknown–and unknowable–at the time of the initial donation. Together, these two facts support the practice of relying on what is variously called blanket, broad, or general consent at the time of donation, in which a person gives open-ended consent to whatever sort of research might later be done. The argument for this approach is that since future uses cannot be known at the time of donation, more specific consent is not possible. Furthermore, because the data used in these studies will be deidentified, there no risk to the donor (setting aside any lingering concerns about re-identification); in fact, under current regulations, there is no longer a human subject involved and thus there is no need for protection.

It is no surprise then that broad consent is widely used [7, 10] and has been endorsed in the proposed revisions to the Common Rule. [11, p. 53974] But members of the public are skeptical about this approach to gaining consent for participation in biobank research. A systematic review of US individuals’ perspectives on broad consent concluded, “many people do not favor broad consent for either research itself or for research and subsequent wide data sharing” [12, p. 7]. The authors of the review explain that while slight majorities supported broad consent *when it was the only choice offered*, when other consent options were available–including “tiered” or “study-by-study” consent–“only a minority of respondents favored broad consent.”

Using data from a nationally representative sample, we take a closer look at public attitudes about broad consent and other approaches to gaining consent for the use of donations to a biobank. In addition to examining the influence of demographic and attitudinal variables on consent policy preferences, we investigate how those preferences are affected by “non-welfare interests” (NWIs). We use this term to describe donor concerns about the moral, cultural, or religious dimensions of research conducted by a biobank. [13–15] These are *not* concerns about risks to the donors’ welfare (such as their privacy interests). Rather, they are concerns about the use of one’s donations for certain types of research—concerns based on the donors’ values and beliefs, such as views about abortion, the use of donations for commerical profit, or xenotransplantation.

## Methods

### Study Population

In June 2014, we surveyed a nationally representative sample of the US population to examine the effect of NWIs on willingness to donate to a biobank using blanket (i.e., broad) consent and on preferences for different policies for gaining consent for the use of donations to a biobank. Participants were recruited from a nationally representative addressed-based internet panel (GfK KnowledgePanel®). GfK sent the survey to 2,654 eligible members of the panel; of those, 1,599 respondents completed the survey (a response rate of 60.2%). Detailed descriptions of GfK’s recruitment and statistical weighting methodologies and the demographics of respondents and non-respondents can be found elsewhere. [16, 17]

The Institutional Review Boards at the both University of Michigan and Michigan State University reviewed the study and determined it to be exempt from federal regulation under exemption #2 of the Code of Federal Regulations, Title 45, Part 46 (http://www.hhs.gov/ohrp/humansubjects/guidance/45cfr46.html#46.101). The dataset is available at http://doi.org/10.3886/E65863V2.

### Survey Measures

At the start of the survey we included a brief introductory description of a fictional biobank, including its function, purpose, and potential benefits for society, as well as a description of “blanket consent.” A variety of terms describe the type of consent under discussion.”Blanket consent” sometimes indicates a completely unregulated consent with no oversight at all, [18] while “broad consent” may refer to consent for unspecified uses with some degree of oversight. [19] However, others use “broad consent” to generically reference any type of consent that covers a spectrum of future uses, including blanket consent. [20–21] In our survey, we defined blanket consent as open-ended consent to any future research projects, all of which would be reviewed by a committee to ensure that the research was of benefit to society and that donor privacy was protected. We used this definition as a way of focusing on the ethical challenges of consenting to future unknown uses of biospecimens, which is the central issue in the conversation about informed consent for biobanking. As a way of measuring respondents’ comprehension, we asked six true/false questions after describing a biobank and defining blanket consent.

As a baseline assessment of participants’ willingness to donate under a blanket consent model, participants were asked to rate their level of agreement or disagreement (on a scale from 1 = Strongly Disagree to 6 = Strongly Agree) with the following sentence: “I would donate tissue samples and medical information to the biobank, so that it can use them for any research study that it allows, without further consent from me.” The survey explained that biobank donations may help with the advancement of medical research but might also be used in research some people could find morally concerning. This explanation was followed by seven (randomly ordered) research scenarios that, according to others [13, 22–26], may be associated with moral concerns (Table 1). In order to measure the potential effect of non-welfare interests (NWI), after each scenario was provided, we assessed the participants’ willingness to provide blanket consent (on the same 6-point scale above), “even if” their donation might be used in the type of research described.

**Table 1:** Research scenarios that may provoke non-welfare interest (NWI) concerns.

Develop more safe and effective abortion methods.
Develop kidney stem cells. The goal would be to grow human kidneys or other organs in a pig that could then be transplanted into people.
Develop patents and earn profits for commercial companies. Most new drugs used to treat or prevent disease come from commercial companies.
Develop stem cells that have the donor’s genetic code. Scientists might use those stem cells to create many different kinds of tissues and organs for use in medical research.
Create vaccines against new biological weapons. The government might need to develop biological weapons of its own when it does this research.
Understand the evolution of different ethnic groups, and where they come from. What they learn might conflict with some religious or cultural beliefs.
Discover genes that make some people more violent. This could lead to ways to reduce violent behavior. But if these genes are found to be more common among some racial and ethnic groups, this might increase prejudice.

We then presented to participants five consent policies ranging from “blanket consent” to “real-time specific” (“study-by-study”) consent (Table 2). We asked respondents to identify whether they found the policy options acceptable or unacceptable and to choose which of the options was the “best” and the “worst” for gaining consent for the use of biobank donations.

**Table 2:** Societal policy options for biobank consent.

Policy Option	Description
Blanket consent	This means that donors have control over whether to donate but not over how the samples are used in any future research. It gives the biobank and researchers a lot of freedom in deciding how to use samples.
Blanket consent combined with a caution	Donors are alerted in advance with the following statement: “Some people may have moral, religious, or cultural concerns about some kinds of research.” Donors can then decide whether they are still willing to donate. Some donors may decide not to donate, resulting in fewer samples for research.
Blanket consent combined with an option to withdraw	Donors first give their blanket consent. The biobank then gives them easy access to information about current research projects being done with donated samples. If donors see research projects that worry them, they can decide to withdraw their tissues. If too many people withdraw their donation, researchers may have trouble finding enough samples to do their research.
Blanket consent combined with limits	Donors are given a short list of types of research projects that might worry some people. The donors then decide which types of research can’t use their donation. Research not on the list would still be covered by a blanket consent. This system may cost more, leaving less money for research.
Real-time specific (study-by-study) consent for use of the donated samples	Donors don’t give blanket consent. Instead, the biobank contacts them and asks for their consent for each specific project. Donors are given maximum control, but some might get tired of being contacted repeatedly. The cost of recontacting every donor for consent will be high. If too many people refuse to give their consent, many research studies will not be possible.

We also collected demographic and attitudinal data, including a measure of “privacy concern,” i.e., how worried respondents would be that an unauthorized person might see their private information, even after being told a “committee will make sure the study…protects your privacy” (on a 5-point scale, 1 = “Not worried at all”, 5 = “Very Worried”), and respondent attitude towards biomedical research in general (using the RAQ–Research Attitudes Questionnaire). [27, 28]

### Analysis

In this paper we explore the the attitudes of respondents toward biobank consent polices. We first report the percentages of respondents identifying each policy as unacceptable or the worst (of the five presented) and then examine the bivariate relationships between these attitudes and respondents’ socio-demographic and attitudinal characteristics. For those policy options described as “unacceptable” or “worst” by a substantial percentage of participants, we used logistic regression to examine how these policy preferences are affected by donor characteristics (socio-demographic and attitudinal). All participant characteristics considered in bivariate analyses were considered as potential predictors of interest.

In order to determine the nature of the relationships–linear or non-linear–between predictors and our outcome variable, we first fit all potential predictors that were continuous or ordinal as categorical dummies, and if the parameter estimates for the categorical dummy variables showed incremental change, we included that variable as a continuous variable. For example, privacy was included in the model as a single continuous variable ranging from not worried at all (1) to very worried (5). Similarly, political affiliation was included as a single variable going from extremely liberal (1) to extremely conservative (7). We dropped predictors that showed no meaningful relationship with any policy option–determined by parameter estimates close to null with corresponding p-values greater than 0.05 –and for consistency in presentation and interpretation, the final model for each policy preference option included the same set of predictors. Adjusted odds ratios (AOR) greater than 1 based on the model indicates that a participant characteristic is positively associated with finding the policy the worst or unacceptable, while controlling for other characteristics in the model.

Finally, we looked at the effect of NWIs on consent policy preference, both in terms of the *type* of NWI—as measured by attitudes toward specific NWI scenarios—and *extent—*as measured by the number of NWI scenarios that respondents found concerning enough to make them unwilling to give blanket consent. Bivariate relationships between each type of NWI (as well as baseline willingness to donate under blanket consent) and the two policy options found to be the worst and the least acceptable (blanket consent and real-time consent) were assessed. In order to analyze the effect of the extent of NWI concerns on policy preferences, we used ANOVA to compare, across each policy option, the mean number of NWI scenarios under which participants were unwilling to donate. For baseline blanket consent and all seven NWI scenarios, we dichotomized their level of agreement into unwilling (scores 1, 2, or 3) and willing (scores 4, 5, or 6). All results, including descriptive statistics, were weighted to correct for stratified sampling designs, non-coverage and non-response.

## Results

The results for the unacceptable and worst policies are presented below (Table 3). Blanket consent was deemed unacceptable by 44% of our sample and was considered the worst option by 38%. The response was similar at the other end of the spectrum with real-time specific consent: 43% found this option to be unacceptable, and 45% found it to be the worst option. Modified versions of blanket consent were less often found unacceptable (ranging from 28% to 35%) and were rarely identified as worst (ranging from 4% to 7%).

**Table 3:** Percent finding societal consent policy “unacceptable” or “worst”.

Policy Option	Unacceptable Option (n = 1,587)^1^ %	Worst Option (n = 1,548)^1^^,^^2^ %
Blanket consent	43.6	37.8
Blanket consent combined with a caution	28.1	4.2
Blanket consent combined with an option to withdraw	29.2	6.2
Blanket consent combined with limits	34.9	6.8
Real-time specific consent for each use of the donated samples	43.0	45.0

^**1**^ Not all respondents answered the question.

^2^ Data previously published in a JAMA research letter at http://doi.org/10.1001/jama.2014.16363.

In order to better understand these policy preferences, we looked at the demographic and attitudinal variables associated with the two policies participants found the most objectionable—blanket consent and real-time specific consent (Table 4). Only a few of the demographic and attitudinal variables were associated with policy preferences, but they are worth noting. Increasing age was associated with finding both blanket and real-time specific consent unacceptable. Respondents who felt abortion should be restricted or who were worried about the privacy of biobank donations had a less favorable view of blanket consent. Trust in research, as measured by the RAQ, was associated with a less favorable view of real-time specific consent. There are no consistent effects of gender, race, education, income, religion, or political views on policy preferences. All socio-demographic data was based on self-report; the racial categories American Indian or Alaska Native, Asian, and Native Hawaiian/Pacific Islander were collapsed into the “Other” category as there were insufficient numbers to properly analyze individually.

**Table 4:** Logistic regression predicting preferences for blanket or real-time specific consent policies.

	Blanket Consent Unacceptable		Real-Time Consent Unacceptable		Blanket Consent Worst		Real-Time Consent Worst	
	OR	p	OR	p	OR	p	OR	p
Age	**1.017**	<0.001	**1.014**	<0.001	1.008	0.053	0.998	0.687
Female	1.085	0.513	1.118	0.326	1.016	0.900	1.053	0.661
White (Ref)								
Black	0.944	0.790	1.319	0.173	**0.612**	0.035	0.852	0.483
Other	1.260	0.315	1.183	0.444	1.184	0.471	**0.621**	0.035
Hispanic	1.139	0.548	1.053	0.801	1.356	0.152	**0.545**	0.005
Education^1^	**1.281**	0.001	1.062	0.368	**1.173**	0.029	1.035	0.628
Income (1–19)	1.001	0.945	0.993	0.602	1.021	0.197	1.015	0.346
Always legal (Ref)								
Legal in most circumstances	1.171	0.385	0.947	0.737	1.181	0.352	0.984	0.928
Legal in a few	**1.431**	0.045	0.837	0.277	**1.833**	0.001	**0.626**	0.007
Always illegal	**2.392**	<0.001	**0.648**	0.043	**2.013**	0.002	**0.594**	0.028
Don't know	1.604	0.109	1.336	0.303	1.624	0.102	**0.446**	0.011
Catholic (Ref)								
Non-Catholic Christian	1.088	0.600	1.023	0.883	1.382	0.050	0.977	0.884
Non-Christian Religion	1.205	0.561	1.139	0.649	1.558	0.164	0.747	0.353
Unaffiliated	1.048	0.808	1.115	0.540	**1.566**	0.020	0.959	0.825
Don't know	1.612	0.206	**2.500**	0.016	**0.360**	0.028	**2.769**	0.010
Privacy worry (1–5; higher = worried)	**1.350**	<0.001	**0.880**	0.012	**1.304**	<0.001	**0.750**	<0.001
Political (1–7; higher = conservative)	1.035	0.449	0.973	0.531	1.016	0.724	1.004	0.928
Cumulative RAQ Score^2^	**0.918**	<0.001	1.005	0.562	**0.939**	<0.001	**1.059**	<0.001

^1^ Ranges from 1–4: “Less than high school” (1), “High school” (2), “Some college” (3), “Bachelor’s degree or higher” (4)

^2^ An 11 item Research Attitudes Questionnaire that assesses attitudes toward medical research. 1–6 Likert scale (Strongly Disagree to Strongly Agree) with cumulative scores ranging from 11–66 (higher is more trusting of medical research).

We next turn to the influence of NWIs on consent policy preferences. We know from our earlier work that NWIs reduce willingness to donate to a biobank using broad consent, [17] and trust in research mediates that relationship: those with more positive attitudes toward research were more willing to donate across all of the NWIs presented. [27] Here we look at the effect of NWIs on consent policy preference, in terms of both *type of NWI–*as measured by attitudes toward specific NWI scenarios–and *extent*—as measured by the number of NWI scenarios in which respondents were unwilling to give blanket consent. Table 5 presents baseline willingness to donate under blanket consent and the simple bivariate relationship between responses to each *type* of NWI scenario and attitudes toward both blanket and real-time specific consent. We found that those who are *not* willing to donate under blanket consent–across all NWI scenarios–have a less favorable view of blanket consent policy in general and are also less likely to think real-time specific consent is the worst option. The type of NWI has little or no effect on policy preferences, as seen in the more or less stable preferences across all NWIs. We found no change in the relationship between type of NWI and policy preference after using multinomial logistic regression to adjust for the socio-demographic and attitudinal variables included in Table 4 (tables available upon request).

**Table 5:** Consent policy preferences by willingness to donate using blanket consent in different types of NWI scenarios and in baseline blanket consent.

	Blanket Consent Unacceptable	Real-time Consent Unacceptable	Blanket Consent Worst	Real-time Consent Worst
**Baseline Blanket Consent**				
Willing	361 (33.4%)	475 (43.9%)	328 (30.7%)	569 (53.3%)
Unwilling	328 (65.7%)	207 (41.3%)	257 (54.1%)	124 (26.1%)
**Abortion**				
Willing	237 (30.2%)	345 (44.1%)	224 (29.1%)	410 (53.2%)
Unwilling	449 (56.7%)	333 (42.0%)	359 (46.7%)	281 (36.6%)
**Kidney Stem Cells**				
Willing	333 (32.6%)	430 (42.2%)	321 (31.8%)	528 (52.2%)
Unwilling	356 (63.5%)	251 (44.7%)	262 (49.3%)	167 (31.5%)
**Patents**				
Willing	272 (31.0%)	361 (41.2%)	253 (29.3%)	463 (53.7%)
Unwilling	416 (59.2%)	321 (45.6%)	330 (48.7%)	231 (34.1%)
**Genetic Code**				
Willing	365 (32.8%)	471 (42.3%)	339 (30.9%)	574 (52.4%)
Unwilling	324 (69.4%)	210 (44.9%)	244 (55.0%)	120 (27.0%)
**Bioweapons Vaccine**				
Willing	286 (31.8%)	364 (40.6%)	272 (30.8%)	458 (51.9%)
Unwilling	400 (58.8%)	316 (46.3%)	310 (47.1%)	237 (36.0%)
**Evolution of Ethnic Groups**				
Willing	327 (32.2%)	438 (43.1%)	302 (30.2%)	522 (52.1%)
Unwilling	363 (64.4%)	243 (43.1%)	281 (51.8%)	173 (31.9%)
**Violence Gene**				
Willing	300 (32.5%)	395 (42.8%)	286 (31.4%)	461 (50.5%)
Unwilling	389 (59.2%)	287 (43.6%)	297 (47.1%)	234 (37.1%)

Table 6 examines the effect of the *extent* of NWI concerns–that is, the *number* of NWIs where a respondent was unwilling to donate using blanket consent–on policy preferences. An increase in the number of concerning NWIs (i.e., an NWI resulting in an unwillingness to donate) is associated with an increased likelihood of finding blanket consent to be the worst policy. This relationship persisted after adjusting for the variables in Table 4 using a multinomial regression (tables available upon request).

**Table 6:** Policy preferences by mean number of concerning NWIs.

Policy judged to be worst	N	Number of concerning NWIs^a^ Mean (SD)
Blanket Consent	583	3.57 (2.32)
Blanket Consent combined with a caution	64	2.65 (2.36)
Blanket consent combined with an option to withdraw	97	3.07 (2.75)
Blanket consent combined with limits	103	2.51 (2.36)
"Real-time" specific consent	695	2.07 (2.15)
Total	1543	2.76 (2.38)

F = 35.065, p < .001

^a^NWI scenarios that respondents found concerning enough to make them unwilling to give blanket consent

## Discussion

Like others [15, 29, 30], we found that members of the public are ambivalent about the use of blanket, or broad, consent for the use of donations to a biobank. Nearly 44% of our nationally representative sample found blanket consent unacceptable and 38% felt it was, in fact, the *worst* in a range of consent policy options. Interestingly, this ambivalence about blanket consent was not accompanied by a desire for real-time specific consent, which was deemed equally unacceptable. In fact, even *more* respondents found real-time specific consent to be the *worst* policy option (45%).

What drives these policy preferences? Those who trust the research endeavor and who have fewer privacy concerns are less likely to favor real-time specific consent. A lack of trust in research and worries about privacy (even when they are told that the samples will be de-identified) push people in the opposite direction. NWIs push people in the direction of wanting the biobank to provide additional cautions about, and control over, how their donations might be used. Those who found blanket consent to be the worst option had, on average, the highest number of NWI concerns.

These findings call for a response on the part of biobanks. And yet, given the nature of biobank research–that it is impossible to know how a specimen may be used in future research at the time of donation–we cannot expect biobanks to develop a model of consent that covers all possible uses and is, at the same time, feasible in terms of cost and timeliness. Nevertheless, and given the key role of trust in promoting cooperation with biobank research, [27] biobanks need to think more broadly about consent and other means of inspiring public confidence about the use of their biospecimens.

For practical reasons, blanket consent must remain, but people need–at a minimum–to be put on notice that their specimens may be used in research they consider to be objectionable. Our data confirm that this information is material to the decision to donate, and thus consent given without this information cannot be considered truly informed. Respect for the moral concerns of donors, however, need not be limited to revising a consent form. For example, and in keeping with the idea of consent as a *process*, biobanks should be transparent with their donors and the general public about supported research, providing up-to-date and readily accessible plain language descriptions of sponsored projects, highlighting potential concerns when appropriate. In addition, donors should be given a way to communicate their questions and concerns to the biobank, and must be and must be given information about how to withdraw their specimens and data from further research uses. Biobanks can also show their respect for the values of donors by giving representatives of the donor community more than token roles in governance, including involvement in decisions about the projects the biobank will support. Treating donors as ongoing partners in the research enterprise will not only build their trust, but the trust of the general public, which is increasingly critical to advances in medical research.

Finally, our results raise concerns about the proposed changes to the Common Rule, which would require at least a blanket consent for most research uses of de-identified *biospecimens*, but not require consent for de-identified *data*. [11] There is no reason to think that people’s moral concerns about uses of their biospecimens would not also extend to uses of their data. In that case, this “biospecimen exceptionalism” [31] is objectionable due to the lack of equal respect for the non-welfare interests of all donors.

Our survey has limitations. While we used a nationally representative internet panel to recruit our respondents, the response rate was a little over 60 percent. Although the response rate presents a challenge to the external validity of our findings, all of the analyses have been weighted to correct for the stratified sampling designs and other sources of survey errors, including non-response and non-coverage. The concise nature of our descriptions of the 5 biobank consent policies and the 7 NWI scenarios may have compromised the internal validity of our findings. We performed significant pilot testing of the survey and came to the conclusion that more detailed descriptions would likely reduce our response rate, as well as increase the variability in how respondents interpreted these descriptions.

## Conclusions

In general, the public is not enamored with blanket, or broad, consent–the most commonly used means of gaining permission for the use of biobank donations. When considered together with other policy options, a substantial minority finds this policy unacceptable. But real-time specific consent is not the answer: here too a substantial minority finds the option unacceptable and labels it the worst among five options. The rejection of these two extremes is good news for biobanks: while continued use of broad consent with no notice of possible objectionable uses of the donations is ethically problematic, members of the public are not asking for the use of costly “study-by-study” consent. The challenge is to find a way to provide potential donors with enough information to allow them to know about, and to retain some control over, the use of their donations. Transparency about sponsored research, together with governance models that assure the donor community and the public that their interests and moral concerns are being respected, will have the felicitous effect of promoting trust in the scientific enterprise, an attribute that is strongly associated with willingness to participate in research and donate to biorepositories.
